# Multiple Chronic Conditions Among US Adults Who Visited Physician Offices: Data From the National Ambulatory Medical Care Survey, 2009

**DOI:** 10.5888/pcd10.120308

**Published:** 2013-04-25

**Authors:** Jill Jacobsen Ashman, Vladislav Beresovsky

**Affiliations:** Author Affiliation: Vladislav Beresovsky, Centers for Disease Control and Prevention, Hyattsville, Maryland.

## Abstract

Most research on adults with chronic conditions focuses on a single disease or condition, such as hypertension or diabetes, rather than on multiple chronic conditions (MCC). Our study’s objective was to compare physician office visits by adults with MCC with visits by adults without MCC, by selected patient demographic characteristics. We also identified the most prevalent dyads and triads of chronic conditions among these patients. We used the National Ambulatory Medical Care Survey, a nationally representative survey of office visits to nonfederal physicians and used 13 of the 20 conditions defined by the National Strategic Framework on Multiple Chronic Conditions. Descriptive estimates were generated and significant differences were tested.

In 2009, an estimated 326 million physician office visits, were made by adults aged 18 years or older with MCC representing 37.6% of all medical office visits by adults. Hypertension was the most prevalent chronic condition that appeared in the top 5 MCC dyads and triads, by sex and age groups. The number of visits by patients with MCC increased with age and was greater for men than for women and for adults with public rather than private insurance. Physicians were more likely to prescribe medications at office visits made by patients with MCC. Physician office visits by adults with MCC were not evenly distributed by demographic characteristics.

## Introduction

Most research on adults with chronic conditions focuses on a single disease or condition, such as hypertension or diabetes; little attention is focused on multiple chronic conditions (MCC) in 1 patient. This study compares, by selected demographic characteristics, physician office visits by adults with MCC with visits by adults without MCC. We also present findings on the most common MCC dyads and triads.

## Analysis

### Data source

The National Ambulatory Medical Care Survey (NAMCS) is a nationally representative annual survey of patient office visits to nonfederal physicians. The NAMCS sampling frame includes all physicians in the American Medical Association and American Osteopathic Association master files, excluding anesthesiologists, radiologists, and pathologists. The 2009 NAMCS (1) used a multistage probability design and included 1,293 physicians who completed patient record forms. The unweighted response rate was 62.1%, and the weighted response rate was 62.4% (1). This study included the 28,693 patient record forms that physicians completed for visits by patients 18 or older for NAMCS 2009. We excluded visits by patients under age 18. Patient information recorded during the visit was abstracted from medical records and entered onto the patient record forms.

Estimates are based on sample data weighted to produce national estimates and include standard errors. Estimates are not presented if they are based on fewer than 30 cases in the sample data. Estimates based on 30 or more cases include an explanation if the relative standard error of the estimate exceeds 30%.

### Definitions

The US Department of Health and Human Services (HHS) Interagency Workgroup on Multiple Chronic Diseases (IWMCD) created a list of 20 chronic conditions common in the United States (2). NAMCS includes check boxes for 13 of these conditions: arthritis, asthma, cancer, chronic kidney disease, chronic obstructive pulmonary disease, congestive heart failure, coronary artery disease, depression, diabetes, hyperlipidemia, hypertension, osteoporosis, and stroke. A checked box for any of these conditions on NAMCS indicates that the medical record contains documentation that the patient was given a diagnosis of the condition at some point, not necessarily during the current visit. The remaining 7 conditions on the HHS-IWMCD list — autism spectrum disease, cardiac arrhythmias, dementia, HIV, hepatitis, schizophrenia, and substance abuse disorders — do not have check boxes on NAMCS. A separate NAMCS question collects the primary diagnosis (as well as 2 additional diagnoses) for the current visit. If 1 of the 7 conditions that are not on the checklist show up in the diagnosis question, we could have included that condition in the count of chronic conditions that we used for this study. However, if the same patient had visited the doctor for a condition or ailment unrelated to 1 of the 7 conditions without check boxes, we would have no way of identifying that chronic condition and could not include it in our count. Because we cannot accurately count the number of visits that patients with these 7 conditions made, we excluded those 7 conditions from our study. We focus exclusively on the 13 conditions with check boxes on NAMCS.

We summed conditions and grouped them into 3 categories (0 or 1, 2 or 3, and ≥4), with MCC defined as 2 or more chronic conditions per visit to a physician. We created dyads by summing yes responses for every combination of 2 chronic conditions. We identified the most common dyads by patients with at least 2 of the 13 chronic conditions (n = 9,871 unweighted visits). We created triads by summing yes responses for every combination of 3 chronic conditions. We identified the most common triads by patients with at least 3 of the 13 chronic conditions (n = 4,986 unweighted visits). In 2009, race data were missing from NAMCS for 24.4% of physician office visits, and ethnicity data were missing for 25.7%. NAMCS staff used model-based single imputation to create imputed race/ethnicity variables. Race/ethnicity imputation is restricted to 3 categories (white, black, and other) on the basis of research by a NAMCS internal work group that identified quality concerns with imputed estimates for race/ethnicity categories other than white and black. Extensive research was conducted on the imputation method and is described in more detail elsewhere (1). We combined the imputed NAMCS race and ethnicity variables to form 4 racial/ethnic groups: non-Hispanic white, non-Hispanic black, non-Hispanic other, and Hispanic persons of any race. “Non-Hispanic other” includes Asians, Native Hawaiian/Pacific Islanders, American Indian/Alaska Natives, and persons of more than 1 race.

NAMCS collects all expected sources of payment listed in the medical chart. We combined these variables into 4 mutually exclusive insurance groups: public insurance (all visits with Medicare or Medicaid/Children’s Health Insurance Program [CHIP]) as the expected source(s) of payment); private insurance (all visits paid for by private insurance, provided the public insurance box was not also checked); and no insurance (visits with self-pay or “no charge/charity” checked, provided that public or private insurance was not also checked); and other (the remaining visits not classified into 1 of the first 3 groups, [visits paid for by Workers’ Compensation and other types of insurance, and unknown: 2.4% of all visits]).

NAMCS collects up to 8 medications that are documented in the medical chart as having been prescribed or provided during the visit. NAMCS includes prescription medications, over-the-counter preparations, immunizations, and desensitizing agents, and medications can be new or continued. Because NAMCS limits the total number of medications that can be recorded per visit to 8, a record of 8 medications indicates that the patient has been prescribed at least 8, but the number could actually be higher. Only 9% of all physician office visits were made by patients with 8 medications, so limiting medications to 8 does not affect a large percentage of total visits (3).

### Statistical analysis

Differences among subgroups were evaluated with 2-tailed *t* tests by using *P* < .05 as the level of significance. A weighted least squares regression analysis was used to evaluate the significance of trends. All comparisons reported in this article were statistically significant.

## Results

### Physician office visits by sociodemographic group

In 2009 adult patients made an estimated 867,783,000 physician office visits (Table 1). Patients with multiple chronic conditions made an estimated 326 million physician office visits representing 37.6% of all visits made by adults (data not shown). The majority of all visits were made by non-Hispanic white adults. Adult visits were distributed evenly by age. An estimated 61.7% of visits were made by women. Approximately half of all visits were made by patients with private insurance and about a third was made by those with public insurance (Table 1). However, there was great variation in insurance status by age. Only 17% and 19% respectively of visits made by those aged 18 to 44 and aged 45 to 64 were made by patients with public insurance, whereas 80% of visits made by those aged 65 and older were made by patients with public insurance (data not shown). Private insurance showed the inverse, with 65% and 71% respectively of visits by those aged 18 to 44 and aged 45 to 64 being made by patients with private insurance, whereas only 17% of visits by those aged 65 or older were made by patients with private insurance (data not shown).

**Table 1:** Selected Demographics of Visits by Patients 18 or Older, National Ambulatory Medical Care Survey, 2009

Selected Demographics	Weighted Number (SE), in 1000s	% (SE)
All patients	867,783 (39,012)	100
**Race/ethnicity**		
Non-Hispanic white	654,544 (33,353)	75.4 (1.3)
Non-Hispanic black	90,797 (6,779)	10.5 (0.6)
Non-Hispanic other^a^	35,273 (5,469)	4.1 (0.6)
Hispanic	87,169 (8,684)	10.0 (1.0)
**Age, y**		
18–44	267,057 (12,331)	30.8 (0.8)
45–64	320,375 (16,670)	36.9 (0.7)
≥65	280,351 (15,021)	32.3 (0.8)
**Sex**		
Women	535,675 (24,422)	61.7 (0.7)
Men	332,108 (16,311)	38.3 (0.7)
**Expected source of payment**		
Private insurance	448,470 (20,736)	51.7 (1.1)
Public insurance^b^	328,782 (19,232)	37.9 (1.1)
Other insurance^c^	53,237 (5,461)	6.1 (0.6)
No insurance^d^	37,294 (2,695)	4.3 (0.3)

a Non-Hispanic other includes Asians, Native Hawaiian/Pacific Islanders, American Indian/Alaska Natives, and persons of mixed race.

b Public insurance includes Medicare and Medicaid.

c Other insurance includes Workers’ Compensation, other insurance, and unknown (2.4% of visits).

d No insurance includes self-pay, no charge, and charity.

### Physician office visits by patients with MCC by sex, age and race

In 2009, 29.2% of physician office visits were made by adult patients with 2 or 3 chronic conditions, and 8.4% of visits were made by patients with 4 or more MCC (Table 2). Visits made by younger patients were less likely to be made by patients with MCC (9.3% of visits by adults aged 18 to 44 years had 2 to 3 chronic conditions compared with 32.4% of visits by adults aged 45 to 64 and 44.4% of visits by patients aged 65 or older). This increasing trend by age for 2 or 3 chronic conditions was seen for visits by men and women and for visits by non-Hispanic white, non-Hispanic black and Hispanic patients. The increasing trend by age for patients with 4 or more chronic conditions was also seen for visits by both sexes and visits by all racial/ethnic groups for which we had reliable estimates.

**Table 2:** Physician Office Visits by Patients With Chronic Conditions by Sex, Age, and Race/Ethnicity, National Ambulatory Medical Care Survey, 2009

Sex, Age, Race/Ethnicity	0–1 Chronic Conditions, % (SE)	2–3 Chronic Conditions, % (SE)	≥4 Chronic Conditions, % (SE)
**Total**			
**All ≥18 y**	62.5 (1.1)	29.2 (0.9)	8.4 (0.5)
Non-Hispanic white	61.4 (1.2)	29.8 (0.9)	8.8 (0.6)
Non-Hispanic black	62.6 (2.1)	30.3 (1.7)	7.1 (0.9)
Non-Hispanic other^a^	71.9 (4.5)	22.3 (3.8)	5.8 (1.3)
Hispanic	66.7 (2.5)	25.9 (1.9)	7.3 (1.6)
**All 18–44 y**	89.7 (0.7)	9.3 (0.6)	1.1 (0.2)
Non-Hispanic white	89.6 (0.7)	9.2 (0.6)	1.1 (0.2)
Non-Hispanic black	87.3 (2.2)	11.6 (2.0)	^—^ b
Non-Hispanic other^b^	92.1 (2.2)	7.6 (2.2)	^—^ b
Hispanic	91.2 (1.3)	7.8 (1.3)	^—^ b
**All 45–64 y**	60.7 (1.1)	32.4 (0.9)	7.0 (0.5)
Non-Hispanic white	60.8 (1.2)	31.8 (1.0)	7.4 (0.7)
Non-Hispanic black	54.5 (2.3)	39.7 (2.1)	5.8 (0.8)
Non-Hispanic other^a^	73.3 (5.7)	22.0 (4.9)	^—^ b
Hispanic	61.0 (3.5)	33.1 (3.4)	5.9 (1.7)
**All ≥65 y**	38.6 (1.3)	44.4 (1.2)	16.9 (1.0)
Non-Hispanic white	39.0 (1.5)	44.3 (1.3)	16.7 (1.0)
Non-Hispanic black	37.0 (3.4)	44.5 (3.1)	18.5 (2.3)
Non-Hispanic other^a^	45.0 (6.1)	41.0 (5.5)	14.0 (3.3)
Hispanic	34.4 (3.2)	46.5 (3.3)	19.2 (4.1)
**Women**			
**All ≥18 y**	65.0 (1.1)	27.0 (0.9)	8.0 (0.5)
Non-Hispanic white	63.5 (1.3)	28.0 (1.0)	8.5 (0.6)
Non-Hispanic black	65.4 (2.5)	27.4 (2.0)	7.1 (1.3)
Non-Hispanic other^a^	75.3 (3.9)	19.2 (3.3)	5.5 (1.5)
Hispanic	70.7 (2.6)	22.5 (2.0)	6.8 (1.6)
**All 18–44 y**	91.0 (0.6)	8.2 (0.6)	0.8 (0.2)
Non-Hispanic white	90.2 (0.7)	8.8 (0.6)	1.0 (0.3)
Non-Hispanic black	90.8 (1.7)	8.6 (1.7)	^—^ b
Non-Hispanic other^a^	94.3 (2.2)	5.7 (2.4)^b^	^—^ b
Hispanic	93.6 (1.3)	6.0 (1.3)	^—^ b
**All 45–64 y**	62.9 (1.2)	30.1 (1.0)	7.1 (0.6)
Non-Hispanic white	62.8 (1.2)	30.0 (1.1)	7.2 (0.7)
Non-Hispanic black	56.4 (2.5)	37.4 (2.5)	6.2 (1.2)
Non-Hispanic other^a^	72.5 (6.1)	20.7 (4.8)	^—^ b
Hispanic	67.1 (3.5)	26.2 (2.9)	6.7 (2.0)
**All ≥65 y**	37.7 (1.6)	44.9 (1.4)	17.4 (1.0)
Non-Hispanic white	38.6 (1.8)	44.4 (1.5)	17.0 (1.2)
Non-Hispanic black	32.7 (3.8)	46.8 (3.7)	20.5 (3.4)
Non-Hispanic other^a^	45.5 (5.8)	41.2 (5.5)	^—^ b
Hispanic	31.6 (3.5)	49.6 (4.2)	18.8 (4.4)
**Men**			
**All ≥18 y**	58.4 (1.3)	32.6 (1.1)	9.0 (0.7)
Non-Hispanic white	58.0 (1.3)	32.6 (1.1)	9.4 (0.8)
Non-Hispanic black	57.1 (3.1)	35.8 (2.8)	7.1 (1.7)
Non-Hispanic other^a^	66.5 (6.7)	27.2 (5.7)	^—^ b
Hispanic	59.4 (3.2)	32.2 (2.7)	8.4 (1.9)
**All 18–44 y**	86.6 (1.3)	11.6 (1.1)	1.8 (0.4)
Non-Hispanic white	88.4 (1.3)	10.1 (1.0)	^—^ b
Non-Hispanic black	77.0 (6.9)	20.1 (5.5)	^—^ b
Non-Hispanic other^a^	85.5 (4.9)	^—^ b	^—^ b
Hispanic	85.4 (2.3)	12.2 (2.2)	^—^ b
**All 45–64 y**	57.5 (1.5)	35.6 (1.4)	6.8 (0.7)
Non-Hispanic white	58.0 (1.6)	34.5 (1.5)	7.5 (0.9)
Non-Hispanic black	51.8 (4.6)	43.0 (4.4)	5.2 (1.5)
Non-Hispanic other^a^	74.4 (6.5)	23.6 (6.1)	^—^ b
Hispanic	51.5 (5.2)	43.9 (5.3)	^—^ b
**All ≥65 y**	40.0 (1.6)	43.7 (1.5)	16.3 (1.4)
Non-Hispanic white	39.5 (1.7)	44.3 (1.7)	16.2 (1.6)
Non-Hispanic black	44.5 (5.8)	40.4 (6.1)	15.1 (4.3)
Non-Hispanic other^a^	44.4 (8.8)	40.7 (8.3)	^—^ b
Hispanic	38.4 (5.1)	41.9 (4.4)	19.7 (4.5)

Abbreviation: SE, standard error.

a Non-Hispanic other includes Asians, Native Hawaiian/Pacific Islanders, American Indian/Alaska Natives, and persons of mixed race.

b Estimate does not meet standards of reliability or precision.

Visits by men (32.6%) were more likely than visits by women (27.0%) to be made by patients with 2 or 3 chronic conditions, and this trend persisted for visits by non-Hispanic whites, non-Hispanic blacks, and Hispanics. For visits by patients younger than 65, visits by men were more likely than visits by women to be made by patients with 2 or 3 chronic conditions. This sex difference persisted for visits by non-Hispanic blacks and Hispanics aged 18 to 44, and for visits by non-Hispanic whites and Hispanics aged 45 to 64. Visits by men and women aged 65 or older were equally likely to be made by patients with MCC, regardless of race/ethnicity.

### Physician office visits by patients with MCC by sex, age, and insurance

We found significant differences among visits made by patients with insurance (public, private, or other) and those with no insurance (Table 3). Visits by patients with private insurance (23.7%), other insurance (20.2%), or no insurance (16.0%) were less likely to be made by patients with 2 to 3 chronic conditions than were visits by patients with public insurance (39.5%). This difference by insurance status persisted for visits made by patients with 4 or more chronic conditions.

**Table 3:** Physician Office Visits by Patients With Chronic Conditions by Sex, Age, and Expected Source of Payment, National Ambulatory Medical Care Survey, 2009

Sex, Age, Expected Source of Payment	0–1 Chronic Conditions, % (SE)	2–3 Chronic Conditions, % (SE)	≥4 Chronic Conditions, % (SE)
**Total**			
**All ≥18 y**			
All sources	62.5 (1.1)	29.2 (0.9)	8.4 (0.5)
Private insurance	71.6 (1.0	23.7 (0.9)	4.7 (0.4)
Public insurance^a^	45.8 (1.6)	39.5 (1.3)	14.7 (0.9)
Other insurance^b^	75.1 (2.6)	20.2 (2.1)	4.7 (0.8)
No insurance^c^	81.4 (2.0)	16.0 (1.7)	2.6 (0.6)
**18–44 y**			
All sources	89.7 (0.7)	9.3 (0.6)	1.1 (0.2)
Private insurance	90.1 (0.8)	9.2 (0.7)	^—^ d
Public insurance a	85.4 (1.6)	11.6 (1.3)	3.0 (0.8)
Other insurance b	93.4 (1.6)	6.3 (1.6)	^—^ d
No insurance^c^	92.5 (1.6)	7.0 (1.5)	^—^ d
**45–64 y**			
All sources	60.7 (1.1)	32.4 (0.9)	7.0 (0.5)
Private insurance	63.0 (1.1)	31.5 (1.1)	5.6 (0.5)
Public insurance a	45.4 (2.8)	40.6 (2.1)	14.1 (1.5)
Other insurance b	67.2 (3.0)	26.6 (2.3)	6.2 (1.2)
No insurance c	69.2 (3.3)	26.5 (2.8)	4.3 (1.1)
**≥65 y**			
All sources	38.6 (1.3)	44.4 (1.2)	16.9 (1.0)
Private insurance	45.3 (2.3)	39.5 (1.8)	15.2 (1.6)
Public insurance a	37.1 (1.4)	45.5 (1.3)	17.4 (1.1)
Other insurance b	32.6 (5.3)	51.3 (5.7)	16.1 (3.9)
No insurance c	75.0 (6.3)	^—^ d	^—^ d
**Women**			
**All ≥18 y**			
All sources	65.0 (1.1)	27.0 (0.9)	8.0 (0.5)
Private insurance	75.1 (1.0)	20.7 (0.8)	4.3 (0.4)
Public insurance^a^	47.4 (1.9)	38.2 (1.5)	14.3 (1.0)
Other insurance^b^	77.8 (2.5)	17.9 (2.1)	4.5 (0.8)
No insurance^c^	82.0 (2.2)	16.0 (2.1)	2.0 (0.5)
**18–44 y**			
All sources	91.0 (0.6)	8.2 (0.5)	0.8 (0.2)
Private insurance	91.3 (0.7)	8.3 (0.7)	^—^ d
Public insurance a	88.1 (1.6)	9.4 (1.3)	^—^ d
Other insurance^b^	94.0 (1.4)	5.5 (1.3)	^—^ d
No insurance^c^	92.9 (1.8)	7.1 (1.8)	^—^ d
**45–64 y**			
All sources	62.9 (1.2)	30.1 (1.0)	7.0 (0.6)
Private insurance	66.1 (1.2)	28.5 (1.2)	5.4 (0.6)
Public insurance^a^	45.9 (2.8)	39.2 (2.3)	14.9 (2.0)
Other insurance^b^	67.6 (3.5)	25.7 (3.1)	6.7 (1.7)
No insurance c	69.4 (3.5)	26.6 (3.3)	3.9 (1.1)
**≥65 y**			
All sources	37.7 (1.6)	44.9 (1.4)	17.4 (1.0)
Private insurance	44.9 (3.4)	38.1 (2.4)	16.9 (2.2)
Public insurance^a^	36.0 (1.7)	46.3 (1.5)	17.6 (1.2)
Other insurance^b^	35.3 (6.5)	50.7 (6.4)	^—^ d
No insurance^c^	79.2 (6.3)	^—^ d	^—^ d
**Men**			
**All ≥18 y**			
All sources	58.4 (1.3)	32.6 (1.1)	9.0 (0.7)
Private insurance	65.9 (1.4)	28.7 (1.2)	5.4 (0.6)
Public insurance a	43.1 (1.7)	41.7 (1.6)	15.2 (1.3)
Other insurance b	71.8 (3.4)	23.1 (3.0)	5.1 (1.4)
No insurance c	80.4 (2.5)	16.1 (2.1)	3.5 (1.2)
**18–44 y**			
All sources	86.6 (1.3)	11.6 (1.1)	1.8 (0.4)
Private insurance	87.2 (1.4)	11.3 (1.2)	^—^ d
Public insurance a	76.3 (3.9)	19.0 (3.0)	^—^ d
Other insurance b	92.4 (2.7)	7.6 (2.7)	^—^ d
No insurance c	92.0 (2.0)	6.9 (1.9)	^—^ d
**45–64 y**			
All sources	57.5 (1.5)	35.6 (1.4)	6.8 (0.7)
Private insurance	58.6 (1.6)	35.7 (1.6)	5.8 (0.7)
Public insurance^a^	44.5 (3.9)	42.7 (3.9)	12.8 (2.0)
Other insurance^b^	66.8 (3.5)	27.5 (3.1)	^—^ d
No insurance^c^	68.8 (4.6)	26.4 (4.1)	^—^ d
**≥65 y**			
All sources	40.0 (1.6)	43.7 (1.5)	16.3 (1.4)
Private insurance	45.9 (2.7)	41.1 (3.1)	13.0 (1.8)
Public insurance^a^	38.5 (1.7)	44.3 (1.5)	17.1 (1.5)
Other insurance^b^	30.4 (6.6)	51.9 (7.6)	^—^ d
No insurance^c^	71.2 (11.5)	^—^ d	^—^ d

Abbreviation: SE, standard error.

a Public insurance includes Medicare and Medicaid.

b Other insurance includes Workers’ Compensation, other insurance, and unknown (2.4% of visits).

c No insurance includes self-pay, no charge, and charity.

d Estimate does not meet standards of reliability or precision.

A greater proportion of visits by patients aged 65 and older with public rather than private insurance were made by patients with 2 to 3 MCC (45.5% and 39.5% respectively). At all ages, a greater proportion of visits by men with public rather than private insurance were made by patients with MCC. Visits made by women aged 45 or older with public rather than private insurance were more likely to be made by women with 2 or more chronic conditions.

Visits made by patients with no insurance were less likely to be made by patients with 2 or more chronic conditions (18.6%) than were visits made by patients with public insurance (54.2%). We saw this same pattern for all ages and for visits by both women and men.

Visits made by men with private insurance were more likely to be made by patients with 2 or 3 chronic conditions (28.7%) than visits made by women with private insurance (20.7%). This sex difference persisted for visits by those younger than 65. We did not find a statistically significant sex difference by private insurance-for visits made by patients aged 65 or older or for visits made by patients with 4 or more chronic conditions.

Visits by men aged 18 to 44 with public insurance were more likely (19.0%) than visits by women aged 18 to 44 (9.4%) with public insurance to be made by patients with 2 or 3 chronic conditions. We did not find a significant sex difference by public insurance for visits made by patients aged 45 or older or for visits made by patients with 4 or more chronic conditions.

### Chronic condition dyads

Hypertension, the most frequently occurring chronic condition, appeared in 21 of the 29 chronic conditions dyads listed in Table 4. The most frequent dyad was hypertension and hyperlipidemia, and its incidence increased with age for women. About 16.6% of visits by women aged 18 to 44 with 2 or more chronic conditions were made by patients having both hypertension and hyperlipidemia. This number increased for visits by women aged 45 to 64 (31.9%) and 65 or older (40.6%). More visits by adult men than women aged 18 to 64 with 2 or more chronic conditions were made by patients with both hypertension and hyperlipidemia. Diabetes with hypertension and diabetes with hyperlipidemia were frequent dyads, appearing in the top 5 dyads for all age groups for visits by both men and women. Hypertension and arthritis was a frequently occurring dyad for visits by both men and women aged 45 or older, and frequency increased with age. Unlike other dyads, visits by women were more likely than visits by men to be made by patients with hypertension and arthritis.

**Table 4:** Physician Office Visits by Patients With the 5 Most Prevalent Dyads of Chronic Conditions, by Sex and Age, National Ambulatory Medical Care Survey, 2009

Sex, Age, and Dyads	≥2 Chronic Conditions^a^, % (SE)
**Women**	
**18–44 y**	
Hypertension/diabetes	18.1 (2.1)
Hypertension/hyperlipidemia	16.6 (2.0)
Depression/asthma	15.6 (2.1)
Hypertension/depression	14.9 (2.0)
Hyperlipidemia/diabetes	11.5 (1.8)
**45–64 y**	
Hypertension/hyperlipidemia	31.9 (1.6)
Hypertension/diabetes	26.1 (1.5)
Hypertension/arthritis	21.3 (1.4)
Hyperlipidemia/diabetes	17.4 (1.3)
Hypertension/depression	15.1 (1.2)
**≥65 y**	
Hypertension/hyperlipidemia	40.6 (1.8)
Hypertension/arthritis	28.6 (2.1)
Hypertension/diabetes	24.0 (1.4)
Hyperlipidemia/arthritis	16.6 (1.6)
Hyperlipidemia/diabetes	14.7 (1.2)
**Men**	
**18–44 y** b	
Hypertension/hyperlipidemia	32.7 (3.8)
Hypertension/diabetes	27.5 (3.4)
Hyperlipidemia/diabetes	22.7 (3.0)
Hypertension/depression	15.2 (2.5)
**45–64 y**	
Hypertension/hyperlipidemia	42.2 (2.1)
Hypertension/diabetes	27.6 (1.8)
Hyperlipidemia/diabetes	20.3 (1.9)
Hypertension/arthritis	15.2 (1.3)
Hypertension/depression	10.3 (1.4)
**≥65 y**	
Hypertension/hyperlipidemia	43.6 (1.8)
Hypertension/diabetes	29.0 (1.4)
Hypertension/arthritis	19.4 (1.6)
Hyperlipidemia/diabetes	19.1 (1.3)
Ischemic heart disease/hypertension	16.3 (1.2)

Abbreviation: SE, standard error.

a The denominator includes all visits by patients with 2 or more CC, (N=337,100,000 visits). The percentage of visits for each age group does not equal 100% because patients may be included in multiple dyads, and data for only the 5 most frequent dyads for each age group are displayed.

b The remaining most frequent dyad estimate for visits by men aged 18–44 y does not meet standards of reliability or precision.

### Chronic condition triads

An estimated 179,518,000 physician office visits were made by patients with 3 or more chronic conditions. Hypertension, the most frequently occurring chronic condition, appeared in every listed triad (Table 5). Hyperlipidemia, the second most frequent chronic condition, was absent from only 5 of the listed triads.

**Table 5:** Physician Office Visits by Patients With the 5 Most Prevalent Triads of Chronic Conditions, by Sex and Age, National Ambulatory Medical Care Survey, 2009

Sex, Age, Triad	≥3 Chronic Conditions^a^, % (SE) a
**Women**	
**18–44 y^b^**	
Hypertension/hyperlipidemia/diabetes	19.9 (5.0)
**45–64 y**	
Hypertension/hyperlipidemia/diabetes	27.4 (2.5)
Hypertension/hyperlipidemia/arthritis	17.5 (2.1)
Hypertension/diabetes/arthritis	14.1 (2.1)
Hypertension/hyperlipidemia/depression	12.2 (1.4)
Hypertension/depression/arthritis	10.8 (1.2)
**≥65 y**	
Hypertension/hyperlipidemia/arthritis	21.8 (2.2)
Hypertension/hyperlipidemia/diabetes	19.3 (1.7)
Osteoporosis/hypertension/hyperlipidemia	11.3 (1.3)
Hypertension/diabetes/arthritis	11.0 (1.1)
Hypertension/hyperlipidemia/depression	10.7 (1.1)
**Men**	
**18–44 y** b	
Hypertension/hyperlipidemia/diabetes	48.9 (6.7)
**45–64 y**	
Hypertension/hyperlipidemia/diabetes	31.5 (2.8)
Ischemic heart disease/hypertension/hyperlipidemia	14.1 (1.6)
Hypertension/hyperlipidemia/depression	10.8 (1.8)
Hypertension/hyperlipidemia/arthritis	9.4 (1.4)
Hypertension/diabetes/arthritis	7.7 (1.3)
**≥65 y**	
Hypertension/hyperlipidemia/diabetes	26.1 (2.0)
Ischemic heart disease/hypertension/hyperlipidemia	17.6 (1.8)
Hypertension/hyperlipidemia/arthritis	15.0 (1.9)
Hypertension/diabetes/arthritis	9.9 (1.3)
Hypertension/hyperlipidemia/cancer	9.3 (1.3)

Abbreviation: SE, standard error.

a The denominator includes all visits by patients with 3 or more chronic conditions, (N=179,518,000 visits). The percentage of visits for each age group does not equal 100% because patients may be included in multiple triads, and data for only the 5 most frequent triads for each age group are displayed.

b The remaining most frequent triad estimates for visits by patients aged 18–44 do not meet standards of reliability or precision.

The most prevalent chronic condition triad was hypertension, hyperlipidemia, and diabetes. This was the only triad for which we could produce reliable estimates for men and women aged 18 to 44. More visits by men (48.9%) than women (19.9%) aged 18 to 44 with 3 or more chronic conditions and more visits by men (26.1%) than women (19.3%) aged 65 and older were made by patients with hypertension and hyperlipidemia and diabetes. There was not a significant sex difference for this triad for those aged 45 to 64. Another common chronic condition triad was hypertension, hyperlipidemia, and arthritis, and more visits by women than men aged 45 to 64 and 65 or older were made by patients with this triad. Hypertension/diabetes/arthritis was also a common triad for visits by patients aged 45 or older. Visits by women aged 45 to 64 (14.1%) were more likely than visits by men aged 45 to 64 (7.7%) to be made by patients with this chronic condition triad. There was not a significant sex difference for this triad for those aged 65 or older. Hypertension/hyperlipidemia/depression was another common chronic condition triad for visits by women aged 45 or older and visits by men aged 45 to 64. We found no difference by sex. Ischemic heart disease/hypertension/hyperlipidemia was a common triad for visits by men aged 45 or older.

### Number of medications ordered or prescribed at visits for patients with MCC

Patients without MCC were more likely to make office visits during which no medications were ordered or prescribed (Figure). For 30% of visits by patients with 0 or 1 chronic condition, no medications were prescribed. This number decreased to about 15% of visits made by patients with 2 to 3 chronic conditions and 11% of visits made by patients with 4 or more chronic conditions. Patients without MCC took fewer medications than did patients with 4 or more. We found a decreasing trend by number of medications for visits by patients without MCC, and an increasing trend for visits by patients with 4 or more chronic conditions. The trend was not significant for visits by patients with 2 or 3 MCC. More medications were ordered or prescribed during visits for patients with MCC. For example, at least 8 medications were ordered or prescribed for 4% of visits made by patients with 0 or 1 chronic condition, but this number climbed to 18% of visits made by patients with 2 or 3 chronic conditions and to 37% of visits made by patients with 4 or more chronic conditions.

**Figure:**
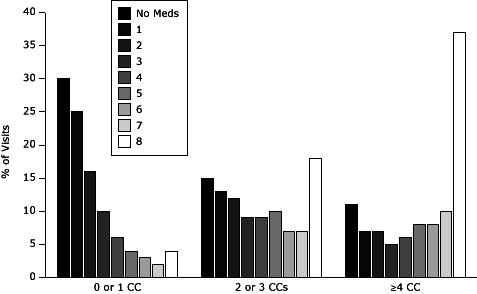
Physician office visits made by patients with or without chronic conditions, by number of medications ordered or prescribed. Shows percentage of office-based ambulatory care visits in which 0 through 8 or more medications were ordered or prescribed for each of 3 chronic condition groupings (0 or 1, 2 or 3 ≥4) (1). **Table:** 

No. of Medications Prescribed	0 or 1 Chronic Condition, %	2 or 3 Chronic Conditions, %	≥4 Chronic Conditions, %
0	30	15	11
1	25	13	7
2	16	12	7
3	10	9	5
4	6	9	6
5	4	10	8
6	3	7	8
7	2	7	10
8	4	18	37

## Summary

Our nationally representative study of office-based ambulatory care visits by adult patients with diagnoses of MCC contributes new information to the field. About 37.6% of adult visits were made by patients with MCC. Visits by patients with MCC were not evenly distributed by demographic characteristics. Visits by women comprised more than 60% of all visits. However, visits by men were more likely than visits by women to be made by patients with MCC. Visits by patients with MCC increased with age and were more likely to be made by patients with public insurance. Hypertension was the most common chronic condition in both dyads and triads. Hypertension-hyperlipidemia was the most common dyad, and these 2 conditions were the most common combination in the top triads, appearing in 17 of the 22 listed triads for this study. The most frequently occurring dyads and triads that included arthritis occurred more often at visits by women than men. However, frequent dyads and triads of other conditions were more likely at visits by men than women. More medications were ordered or prescribed during visits by patients with MCC than by patients without MCC. Visits by patients without MCC were more likely to have no or fewer medications ordered or prescribed than visits by patients with 4 or more chronic conditions.

Our study has limitations. Because the unit of analysis for NAMCS 2009 is an ambulatory care visit to a physician in the United States, the number of visits rather than number of people are measured, so it is possible for the same person to be counted multiple times. In addition, anyone who did not visit a doctor in 2009 was excluded from NAMCS 2009, including Medicare beneficiaries who did not visit a doctor. Thus, our results are not directly comparable with results presented in the *Chronic Conditions Among Medicare Beneficiaries, Chart Book* (4). According to the chart book, about 16% of all Medicare beneficiaries did not visit a doctor in 2009, representing about 33% of beneficiaries without MCC and about 9% of beneficiaries with MCC (4). The National Health Interview Survey (5) estimates that about 92% of US residents aged 65 or older have Medicare (5). NAMCS 2009 estimated that 79% of visits by patients aged 65 or older were made by patients with Medicare. NAMCS has check boxes for 13 chronic conditions, whereas the chart book tracks 15 chronic conditions (4); therefore, our results may undercount Medicare beneficiaries with MCC. Despite these differences, we found that visits by patients with MCC were more likely to be made by patients with public rather than private insurance.

As a nationally representative survey of office-based medical care, our study provides information about ambulatory medical care received by patients with MCC by demographic characteristics and identified the most frequently occurring dyads and triads of chronic conditions.
